# Preparation and biological properties of a novel composite scaffold of nano-hydroxyapatite/chitosan/carboxymethyl cellulose for bone tissue engineering

**DOI:** 10.1186/1423-0127-16-65

**Published:** 2009-07-14

**Authors:** Jiang Liuyun, Li Yubao, Xiong Chengdong

**Affiliations:** 1 Chengdu Institute of Organic Chemistry, Chinese Academy of Sciences, Chengdu 610041, PR China; 2 Research Center for Nano-Biomaterials, Analytical and Testing Center, Sichuan University, Chengdu 610064, PR China

## Abstract

In this study, we report the physico-chemical and biological properties of a novel biodegradable composite scaffold made of nano-hydroxyapatite and natural derived polymers of chitosan and carboxymethyl cellulose, namely, n-HA/CS/CMC, which was prepared by freeze-drying method. The physico-chemical properties of n-HA/CS/CMC scaffold were tested by infrared absorption spectra (IR), transmission electron microscope(TEM), scanning electron microscope(SEM), universal material testing machine and phosphate buffer solution (PBS) soaking experiment. Besides, the biological properties were evaluated by MG63 cells and Mesenchymal stem cells (MSCs) culture experiment *in vitro *and a short period implantation study *in vivo*. The results show that the composite scaffold is mainly formed through the ionic crossing-linking of the two polyions between CS and CMC, and n-HA is incorporated into the polyelectrolyte matrix of CS-CMC without agglomeration, which endows the scaffold with good physico-chemical properties such as highly interconnected porous structure, high compressive strength and good structural stability and degradation. More important, the results of cells attached, proliferated on the scaffold indicate that the scaffold is non-toxic and has good cell biocompatibility, and the results of implantation experiment *in vivo *further confirm that the scaffold has good tissue biocompatibility. All the above results suggest that the novel degradable n-HA/CS/CMC composite scaffold has a great potential to be used as bone tissue engineering material.

## Background

Nowadays, bone tissue engineering, using a porous scaffold material to induce the formation of bone from the surrounding tissue or to act as a template to grow cell for bone tissue regeneration, has some distinct advantages over autografting and allografting[1], and it is a rapidly growing alternative approach to heal damaged bone tissue. However, in the realm of bone tissue engineering, it is a great challenge to develop a desirable porous scaffold material used for successful bone regeneration. Obviously, an ideal scaffold for bone tissue engineering should have highly interconnected porous structure, good mechanical property and biocompatibility[2,3]. In addition, to achieve the requirements better for bone regeneration, biomimetic matrices are usually adopted, which may provide a suitable microenvironment to promote osteoblast proliferation and osteogenesis[4]. As we know, the extracellular matrices (ECMs) of hard tissue are composed of organic and inorganic phases, the inorganic phase consisting primarily of nano-hydroxyapatite(n-HA)crystals, and the organic phase consisting mainly of type I collagen and small amount of ground substance including glycosaminoglycans (GAGs), proteoglycans and glycoproteins[5]. So n-HA/polymer composite scaffolds have been reported in succession during the process of designing the scaffold for bone tissue engineering, such as, n-HA/collagen [6], n-HA/gelatin[7], n-HA/polyamide[8,9], n-HA/poly(L-lacticacid)[10], n-HA/poly(lactide-co-glycolide)[11], which are designed according to bionic principle. However, among the selected polymers, natural biodegradable polymers are a kind of promising candidate, because they avoid a second surgical operation after new bone tissue regeneration, and they possess better biocompatibility and lower cost than synthetic polymers.

Chitosan(CS), a deacetylated derivative of chitin, is a biodegradable and biocompatible cationic natural polymer, and CS-based materials can accelerate bone formation because of the similarity to GAGs in structure [12-15]. Consequently, n-HA/CS composite scaffold has been widely studied for bone tissue engineering [16-20]. However, a poor interaction exists between n-HA and CS phases so that the n-HA/CS composite scaffold has poor physico-chemical properties. Fortunately, carboxymethyl cellulose(CMC) is a natural biodegradable and biocompatible anionic polymer obtained from natural cellulose by chemical modification, and it is very similar to CS in structure, thus, there is strong ionic cross-linking action between CMC with CS[21,22].

Based on the above thought, in this paper, CMC was first introduced into n-HA/CS system, where n-HA was expected to be incorporated into the polyelectrolyte of CS/CMC so as to has a strong interaction between n-HA and polymer[23], accordingly, a novel n-HA/CS/CMC composite scaffold was also fabricated. In addition, to investigate the potential of the novel biodegradable n-HA/CS/CMC composite scaffold to be used for bone tissue engineering material, the physico-chemical properties including the porous morphology, compressive strength, structural stability and degradation *in vitro *and the microstructure of n-HA/CS/CMC composite scaffold were investigated. Besides, its biological properties such as cell biocompatibility *in vitro *and tissue biocompatibility *in vivo *by a short period implantation were also preliminarily studied. The main purpose of the study is to make full use of the most abound natural derived polymer of cellulose and chitin, to develop a novel biodegradable composite scaffold for bone tissue engineering material according to the bionic principle.

## Materials and methods

### Materials

CS powder(Mw2.5 × 10^5^, 80% degree of deacetylation) was purchased from Haidebei Bioengineering Co. Ltd, Jinan, China. CMC-Na(Mw 4.2 × 10^8^, substitution degree of 0.7) was purchased from Kelong Chemical Agent Factory, Chengdu, China. n-HA slurry was prepared by our group[24]. Other reagents used here were all of analytical grade.

### Methods

#### Preparation of n-HA/CS/CMC composite scaffold

The n-HA/CS/CMC scaffold was fabricated as described by the following procedure. Firstly, 3 g CS and 3 g CMC powders were mixed evenly and added into 150 ml n-HA slurry(containing 4 g n-HA powder)with constant stirring for 2 hours under ambient conditions to obtain a homogeneous mixture. Secondly, 3 ml glacial acetic acid was added, and the stirring was kept till solidified mixture was obtained. Then the solidified mixture was maintained in a freezer at -30°C for overnight to freeze the solvent. Finally the sample was lyophilized in a freezing dryer until dried, and a scaffold was achieved. To remove the residue acetic acid, the scaffold was immersed in 0.2 mol/l NaOH solution for several hours, and then washed in deionized water and dried in a vacuum oven at 60°C.

#### Fourier transform-infrared (FT-IR) spectroscopic studies

Infrared spectroscopy was used to characterize intermolecular interaction between components in system. The IR spectra of n-HA, CS, CMC and n-HA/CS/CMC composite scaffold were recorded with a FT/IR spectrophotometer(American Perkin Elmer Co.) by using a KBr die kit. The spectra were collected over the range of 4000-400 cm^-1^.

#### Transmission electron microscope(TEM) observation

The microstructure of n-HA/CS/CMC composite scaffold was examined with transmission electron microscope(TEM), (JME-100CX, Seike Instruments) on a 200 kV. TEM samples were prepared by ultrasonication dispersion method using deionized water.

#### Scanning electron microscope (SEM) observation

The surface morphology of n-HA/CS/CMC composite scaffold was examined with scanning electron microscopy(SEM). The scaffold was gold-coated and observed with SEM (JSM -5900LV, Japan) at an accelerating voltage of 20 kV.

#### Mechanical property test

The compressive strength of the n-HA/CS/CMC scaffold was tested using universal material testing instrument (AG-10AT, DaoJin, Japan) following the guideline of ASTM standard D 695–96. Three parallel samples of the scaffold were cut into cylindrical blocks with a size of Φ6 × 12 mm and conducted with a constant strain rate of 5 mm min^-1 ^until 50% reduction in specimen height, and the mean value of the compressive strength was given.

#### Porosity measurement

The porosity was determined by the liquid displacement method[25]. Briefly, the specimen was immersed into the dehydrated alcohol for 48 h until it was saturate, and the porosity of the sample was calculated according to the formula of P = (W_2_- W_1_)/(ρV_1_), where W_1 _and W_2 _represent the weight of the sample before and after immersing, respectively, and V_1 _is the volume before immersing, ρ is a constant of the density of dehydrated alcohol. Three parallel samples of the scaffold were conducted.

#### In vitro soaking test

The structural stability and degradation were investigated by phosphate buffer solution (PBS) soaking. The n-HA/CS/CMC scaffold samples were dried and weighed, noted as W_0_. The samples were immersed in tube containing 10 ml of PBS, kept oscillating at 37.0 ± 0.5°C. After soaking for 5, 10, 15, 25 and 30 days, the samples were withdrawn from the solution, gently rinsed with deionized water and weighed after being dried, and noted as W_1_. The rate of weight loss (W_L_) was calculated according to the formula of W_L _= (W_0 _- W_1_)/W_0 _× 100%. Five parallel samples of the scaffold were also carried out.

#### In vitro cell culture experiments

To evaluate the cell biocompatibility of the scaffold, MG63 cells and mesenchymal stem cells (MSCs) were both investigated. MG63 cells were human osteosarcoma osteoblasts, obtained from Center for Cell Culture in Wuhan University. MSCs were isolated from neonatal Wista rats calvaria by the sequential enzymatic digestive process and placed in a standard culture medium containing Dulbecco's Modified Eagle medium (D-MEM) (10% fetal bovine serum (FBS), 200 mg/ml penicillin, and 200 mg/ml streptomycin), then MSCs at passage three were removed into the culture medium containing osteogenic reagents(50 mg/l L-ascorbic acid, 10^-8 ^mol/l dexamethasone and 10 mmol/l b-glycerophosphate, 10 mmol/l VitD3, 100 mg/ml penicillium and100 mg/ml streptomycin, 0.3 mg/ml amphotericin, 2.2 g/l sodium bicarbonate and 10% fetal bovine serum). The medium was changed every 2 days [26].

The n-HA/CS/CMC composite scaffolds with a size of 2 × 10 × 12 mm^3 ^were sterilized using ethylene oxide gas and placed in a 12-well cell culture plate. Approximately 2 × 10^5 ^cells of MG63 or MSCs were seeded on the scaffold with undisturbed in an incubator for 3 h, then an additional 1 ml of culture medium was added into each well, and the scaffold/cell samples were cultured in an incubator(37°C with a humidified 5% CO_2 _atmosphere) for 11 days. Medium was changed every 2 days. The controls(empty wells without scaffold) were treated in the same manner.

Estimation of cellular growth was accomplished using the MTT(3–4,5 -dimethylthiazol-2yl{-2,5-diphenyl-2H-tetrazoliumbromide)assay. The medium in the cell-loaded scaffold culture plate was removed after cultured for 1, 4, 7 and 11 days, and 2 ml MTT solution was added to each sample. Following 4 h incubation at 37°C in an air atmosphere containing 5% CO2, and DMSO was used to dissolve the formazan crystals, and the optical densities(OD) were determined using an Elisa microplate reader (ELx 800, BIOTEK) at 570 nm comparied with DMSO blank, which is linear correlations to cell numbers[27]. At least 4 wells were randomly examined each time. The controls(empty wells without scaffold) were treated in the same manner.

#### In vivo implantation

Twelve skeletally mature female adult SD rats(approximately 2 months old and weighing 200 g) were anesthetized with pentobarbital sodium. After the skin was prepped and sterilized with iodine, a ~2 cm incision was made on the lateral thigh of the rat. The gluteus maximus muscle was exposed and an incision of ~1.5 cm was cut on the muscle to make a small pouch. The n-HA/CS/CMC composite scaffold with 4 × 8 mm^2 ^in size was implanted into the muscle pouch. After 2 and 4 weeks implantation, six rats were sacrificed every time, and the scaffold samples were harvested. The three specimens were fixed with 4% formaldehyde for 4 d, then dehydrated in 50%, 75%, 85%, 95%, and 100% ethanol and embedded with paraffin. The specimens were cut into slices of 5 μm in thickness and stained with H&E and Masson's trichrome for the observation by light microscopy (Olympus, Japan). Meanwhile, the scaffold morphology of the other three specimens were observed by SEM after 2 and 4 weeks implantation.

## Results

### Physcio-chemical properties of n-HA/CS/CMC composite scaffold

**Figure 1:**
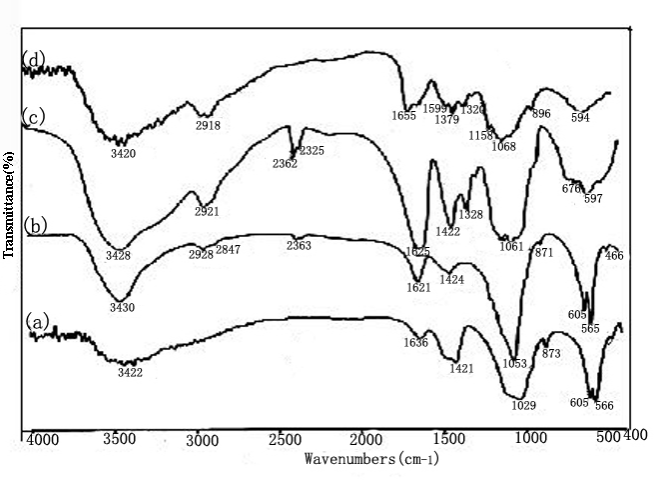
**IR spectra of a)pure n-HA, b) n-HA/CS/CMC, c) pure CMC and d) pure CS**.

**Figure 2:**
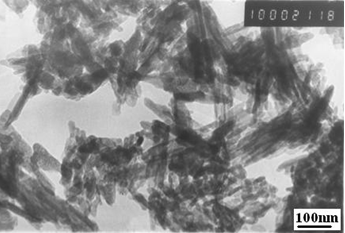
**The TEM photograph of n-HA/CS/CMC composite**.

In order to illustrate intermolecular interaction between components in system, IR spectroscopy measurements were taken (Fig. 1). Comparing the IR spectra of pure n-HA, CS, CMC and n-HA/CS/CMC composite scaffold, it can be found that the specific peaks of pure n-HA, CMC and CS all appeared in the spectrum of n-HA/CS/CMC composite scaffold as shown in Fig. 1b, which suggests that there is no change of the three compositions after compounding. However, an absorption at 1655 cm^-1 ^in CS were shifted to 1621 cm^-1 ^in n-HA/CS/CMC composite scaffold, and the peak of -NH_2_(1599 cm^-1^) was absent, which may be the result of the formation of -NH_3_^+^, and the peak of asymmetry stretching of -COO^- ^is still found at ~1420 cm^-1^. Obviously, these observations mean that there was electrostatic attraction between -NH_3_^+ ^of CS and -COO^- ^of CMC, which results in the formation of CS/CMC polyelectrolyte network, and n-HA was filled in it easily. Additionally, to further demonstrate the microstructure of distribution state of n-HA in composite, TEM photograph of the n-HA/CS/CMC composite was given (Fig. 2). It shows that n-HA crystals are still in the range of nanometer grade and have a good dispersive property in the polymers, which has a positive effect on the mechanical and biological properties of the n-HA/CS/CMC composite scaffold.

**Figure 3:**
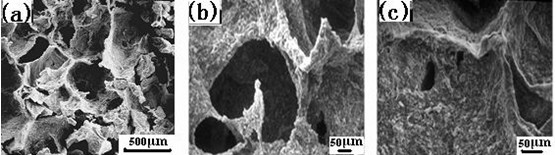
**The SEM microstructure of n-HA/CS/CMC composite scaffold**.

The SEM images (Fig. 3) showed that the scaffold was three-dimensional complicated irregular porous structure together with good interconnections between the pores(Fig. 3(a) and 3(b)), and the walls of the macropores ranging about from 100 to 500 μm contained many micropores(Fig. 3(c)). In addition, the porosity was 77.8% ± 3.24. More important, the compressive strength of the scaffold could also reach 3.5 ± 0.13 MPa, which was in the range of cancellous bone(2 MPa~10 MPa)[28]. Based on the highly porous structure, we thought that it was likely for the scaffold to promote cell adhesion and attachment as well as nutrient delivery to the site of tissue regeneration[29,30].

**Figure 4:**
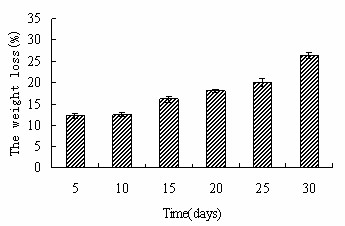
**The weight loss of n-HA/CS/CMC composite scaffold as the function of time in PBS**.

After PBS soaking for 30 days, we find that the scaffold still remain the original shape, which indicates that the scaffold has good structural stability. In addition, Fig. 4 gives the weight loss of n-HA/CS/CMC scaffold as a function of soaking time in PBS. According to the weight loss tendency, we found that the weight loss increased gradually, and the weight loss was tested as near 30% after 30 days soaking, which showed that the scaffold degraded with the soaking time.

### In vitro cell attachment and proliferation

**Figure 5:**
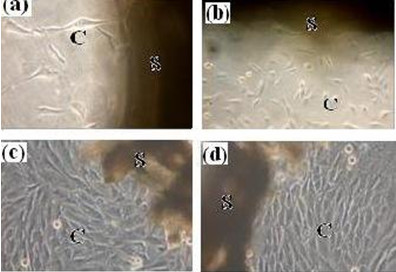
**Phase-contrast microscopy photographs of MG63 and MSCs on the n-HA/CS/CMC composite scaffold after in vitro culture for different days (a) 4 day for MG63, (b) 7 days for MG63, (c) 4 days for MSCs, (d) 7 days for MSCs**. (S, scaffold; C, cells).

**Figure 6:**
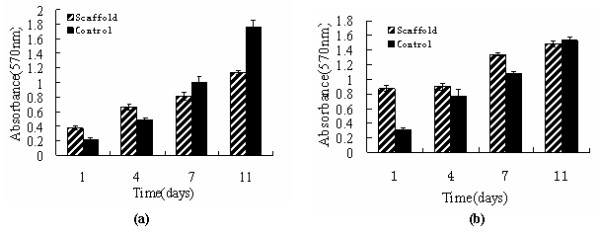
**MTT assay for proliferation of MG63 and MSCs on the n-HA/CS/CMC composite scaffold after in vitro culture for different days(a) MG63, (b) MSCs**.

Fig. 5 shows the morphology of the cells attachment and spreading on the scaffold for different days, observed by phase-contrast microscopy. At 4 days, MG63 cells attached on the surface and spread well. After 7-days culture, more and more cells attached tightly with their filopodium and lamellipodium, and elongated to every corners of scaffold(Fig. 5(b)). Similarly, more MSCs adhered on the scaffold than MG63 after culturing for 4 days. And at the seventh day, cells have dramatically proliferated and aggregated each other to form stratified cell layers on the surface(Fig. 5(d)), indicating the scaffold was nontoxic and suitable for the attachment and growth of both MG63 and MSCs. The cell proliferation on the scaffold was assessed using MTT test. Fig. 6 gives OD value after 1, 4, 7 and 11 days of culture. According to the data, it can be found that both MG63 and MSCs increased with time during the *in vitro *culture period, and the scaffold group has obvious proliferation tendency, suggesting the scaffold did not retarded the cell proliferation, which shows the scaffold is nontoxic.

Based on the above results, we took it for granted that the novel n-HA/CS/CMC composite scaffold had good cell biocompatibility *in vitro*.

### In vivo tissue biocompatibility

**Figure 7:**
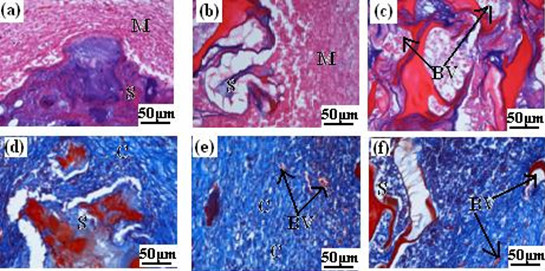
**Histological sections optical micrographs of n-HA/CS/CMC composite scaffolds harvested at different times after implantation**. Scaffolds stained with H&E at (a) 2 weeks, (b) and (c) 4 weeks. Scaffolds stained with Masson's trichrome at (d) 2 weeks, (e) and (f) 4 weeks. (S, scaffold; M, muscle; C, collagen; BV, blood vessels.)

Tissue biocompatibility *in vivo *of the scaffold was evaluated by a short time implantation in muscles of rats and harvesting after 2 and 4 weeks. During the experiment period, all rats remained good health, and the surgical incisions healed well without any wound complications. After 2 and 4 weeks harvest, the histological sections of the scaffold specimen were stained with H&E and Masson's trichrome, respectively (Fig. 7). According to Fig. 7(a)–(c) (stained with H&E), we can find that the muscle cells were normal and there was no evident foreign body reactions for the surrounding tissues at 2-weeks implantation(Fig. 7(a)). After 4-weeks implantation, it could be seen that the scaffold integrated well with surrounding tissues and there was no visible interface between muscles (M) and the scaffold (S) (Fig. 7(b)). Meanwhile, it can be found that most of the scaffold had been biodegraded and many blood vessels(BV) had grown into the pores of the scaffold (Fig. 7(c)). Fig. 7(d)–(f) were the histological sections stained with Masson's trichrome, which were used to assess the formation of collagen and vascularization. After 2 weeks implantation, there was bulk collagen (marked with letter C) in blue appeared (Fig. 7(d)), after 4 weeks, most surface of the scaffold was covered by collagen, indicating that the scaffold had good biocompatibility(Fig. 7(e)). Additionally, a large number of small blood vessels were also seen in the scaffold(Fig. 7(f)), which were favorable to deliver nutrient and ensured cells to survive and proliferate so as to construct new tissues.

**Figure 8:**
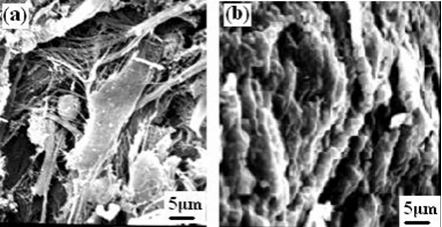
**The SEM microstructure of n-HA/CS/CMC composite scaffold harvested at different times after implantation in muscle (a) 2 weeks, (b) 4 weeks**.

In addition, to make a further investigation on the biocompatibility *in vivo*, the SEM microstructure of the scaffold sample harvested after 2 and 4 weeks implantation in body were given(Fig. 8). It also showed that many cells had grown into the pores of the scaffold after 2 weeks implantation. Likewise, more and more new tissues almost covered the pores at 4 weeks of implantation (Fig. 8(b)), indicating that the scaffold had good tissue biocompatibility.

## Discussion

In order to achieve successful regeneration of damaged bone based on the tissue engineering concept, it is critical to select the proper component to develop a scaffold. In this study, we chose n-HA, CS and CMC as raw materials, which all have good qualities such as biocompatibility, bioactivity, biodegradation. Moreover, it is designed according to the bionic principle. On the other hand, there has a closely relation between physico-chemical properties of a scaffold and the structure of the component in itself. In this paper, CS and CMC have similar structure and opposite electric charge, which can interact strongly and form polyelectrolyte net structure by static electric interaction in adequate acidity solution, simultaneously, n-HA was incorporated into the polyelectrolyte matrix. Thus, we can obtain the n-HA/CS/CMC composite in the form of solidified mixture without adding any other cross-linking agents under ambient conditions, subsequently, we can achieve the porous scaffold by freeze-drying method, which is incomparable for other system scaffold in the same conditions, such as n-HA/CS scaffold, n-HA/CS-Gel[31].

In addition, the scaffold had good three-dimensional irregular porous network structures with pore size of 100~500 μm. These macropores can promote the formation of internal mineralized bone, and the micropores and the interconnected pores were all conduce to nutrient delivery. Moreover, the original compressive strength of 3.5 MPa with a porosity of 77.8%. Obviously, it can bear the reconstruction of new bone tissue. On the other hand, biodegradable scaffold has a great superiority. Here, CS and CMC are both biodegradable polymers, theoretically, the n-HA/CS/CMC composite scaffold is a degradable scaffold. In this study, we chose PBS as the soaking solution, a suitable and reliable method, to investigate the degradation and structural stability. According to the results of the soaking in PBS, it can be concluded that the composite scaffold is degradable. Moreover, it has good structural stability after soaking 30 days, which is also contributed to the intermolecular interaction between components in system and microstructure.

In a word, the novel degradable n-HA/CS/CMC composite scaffold prepared here had good physico-chemical properties, and it was suitable to act as a template to grow cell for bone tissue regeneration.

It is no doubt that the scaffold used for bone tissue engineering should be non-toxic and have good cell biocompatibility, which is a central criteria to ultimately decide the feasibility of implantation in body. The information of the cell attachment and proliferation on the scaffold provided by cell culture experiment *in vitro *is often used as an important initial evaluation of cell biocompatibility. In the present study, MG63 and MSCs are used in the cell seeding experiment, the phase-contrast microscopy observation and the MTT assay results show the n-HA/CS/CMC scaffold is nontoxic and cell biocompatible, which is contributed to the ideal three-dimensional porous structure and mechanical property. However, the chemical composition and the method of fabrication of the scaffold play a more important role in cell biocompatibility[32]. In this paper, n-HA, CS and CMC are all nontoxic and hydrophilic. Moreover, comparing with other methods to develop porous scaffold, such as particulate leaching[33] or foaming method[34], the method here avoided using any poisonous solvents in the process of preparation. Therefore, the scaffold is cell biocompatible, and it is suitable for implantation.

Although *in vitro *test has given some preliminary indicatives of cell biocompatibility, it is still necessary to procure a much clearer idea of host tissue response to the scaffold after implantation *in vivo*. In this study, the scaffold was placed in the muscle of rats for 4 weeks. For the short period implantation, from the roughly observation, we find all experimented rats survival before harvesting, which shows that the sample of implantation is nontoxic and no evident feign body reaction. By observing histological sections, we find that most of the scaffold had been biodegraded after 4 weeks, which results from the biodegradable component of CS and CMC, and it is useful to blood vessels and new tissues to ingrow. Moreover, the tissue had no obvious inflammation, which shows the scaffold in itself and degradation products are all nontoxic. In addition, most of the scaffold was covered by collagen, suggesting the scaffold had osteoinduction to some extent due to the biomimetic matrices of n-HA/CS/CMC composite scaffold. On the other hand, we observed the microstructure of samples by SEM after 2 and 4 weeks, the results also showed that various types of cells and tissues grew into the pores, even covered the scaffold, which is another evidence to demonstrate the tissue biocompatibility of the scaffold.

In conclusion, the results of cell culture experiment *in vitro *and a short period of implantation *in vivo *indicated that the scaffold had good biocompatibility, biodegradation and osteoinduction to some extent. All these results showed that the scaffold can meet the requirement of biological properties for bone tissue engineering.

## Conclusion

In this paper, a novel degradable n-HA/CS/CMC composite scaffold was developed by freeze-drying method. Based on the above analyses and discussion, it can be concluded that the scaffold had desirable physico-chemical properties due to the strong ionic cross-linking interactions between CS and CMC, such as highly complicated interconnection irregular porous network structure(the pore size ranging about from 100 to 500 μm), and the compressive strength reached 3.5 MPa with the porosity of 77.8%, which would meet the basic requirement to grow for new bone tissue. In addition, the MG63 and MSCs cells attached and proliferated well on the scaffold during cell culture period *in vitro*, and the short period implantation experiment *in vivo *further confirmed that the scaffold had no feign body reaction and many blood vessels grew into the porous structure while the scaffold was biodegraded gradually. Meanwhile, most scaffold surface was covered by collagen after 4 weeks implantation, which shows the scaffold had good tissue biocompatibility. In conclusion, the above results indicated that n-HA/CS/CMC composite scaffold was a novel biodegradable porous material with a desirable physico-chemical and biological properties to meet the essential requirement for bone tissue engineer materials, suggesting a potential applications in the field of bone tissue engineer, which is also a new approach to exploit the most abundant natural resource of cellulose and chitin.

## Competing interests

The authors declare that they have no competing interests.

## Authors' contributions

LY and XC conceived of the study, and participated in its design and coordination. All authors read and approved the final manuscript.
